# Feasibility and Acceptability of a Ugandan Telehealth Engagement Platform for Informational Messaging on Modern Contraception: Pilot Cross-sectional Study

**DOI:** 10.2196/34424

**Published:** 2022-06-28

**Authors:** Louis Henry Kamulegeya, JohnMark Bwanika, Joy Banonya, Joan Atuhaire, Davis Musinguzi, Vivian Nakate, Joshua Kyenkya, Lydia Namatende, Keith J Horvath, Agnes Kiragga

**Affiliations:** 1 The Medical Concierge Group Projects and Research Department Kampala Uganda; 2 Infectious Diseases Institute Makerere University Department of Research Kampala Uganda; 3 Department of Psychology San Diego State University San Diego, CA United States

**Keywords:** telehealth, mHealth, digital health, family planning, contraception, messaging, male involvement, health education, Uganda

## Abstract

**Background:**

With the region’s highest population growth rate (30%), Uganda is on the brink of a population explosion, yet access to and utilization of public health control measures like modern contraception is a challenge. This is due to remotely located health facilities, noncustomized health content, and poor or nonfunctional post-facility follow-up.

**Objective:**

The aim of our study was to evaluate the feasibility and acceptability of a telehealth engagement platform primarily targeting men; the platform provided behavioral and informational messaging on modern contraception (ie, family planning) and its impact on shaping sexual and reproductive health and knowledge and uptake of family planning services.

**Methods:**

A longitudinal cohort of men aged 18 years and older gave consent to receive mobile phone messages on family planning; follow-up was performed at months 1, 4, and 6 to assess key study-related outcomes on knowledge transfer and acquisition on modern contraception, partner communication, and spousal uptake of family planning. Qualitative interviews with the study participants’ spouses were also performed.

**Results:**

The study included 551 study participants, 450 of whom were men, the primary study participants, who received the family planning mobile messages and 101 of whom were their spouses. Of the 450 primary participants, 426 (95%) successfully received the messages and only 24 (5%) reported not receiving them. The average response (ie, participation) rate in weekly quizzes was 23%. There was a noted 18.1% increase in couple communication attributed to the intervention; couples opened up more to each other on matters concerning family planning.

**Conclusions:**

Using digital channels to address the concerns and inquiries of participants in real time or as fast as possible helped to increase the likelihood that couples adopted family planning.

## Introduction

### Background

In 2021, Uganda’s population was 42.4 million people, representing growth of about 30% compared to 2014, when the national population census reported a population of 36.9 million [1]. Over 55% of the country’s population was below the age of 15 years and thus close to childbearing age.

According to the 2016 Uganda Demographic Health Survey, the total average fertility rate was 5.4 children per woman [2]. However, the rate is known to be higher in rural and semiurban parts of the country, where on average a woman gives birth to 7 children in her lifetime, making Uganda a country with one of the fastest growing populations in the world. These trends point to a looming population explosion in a setting with high poverty levels, low literacy rates, and limited access to quality health services unless population mitigation measures are urgently taken up.

Population explosion control measures, such as modern contraception, have shown promise in driving socioeconomic growth and political stability in sub-Saharan Africa [3,4]. However, modern contraception (ie, family planning) uptake in Uganda had tended to remain suboptimal, with a 30% contraceptive prevalence rate; the current unmet need for family planning among women has been placed at 34% [5,6].

A number of factors have been identified to account for the above trends, including a lack of access to credible information on modern contraception, lack of male partner support and engagement in decision-making, and poor or nonfunctional post-service follow-up mechanisms to address challenges like side effects, myths, and misconceptions [7,8].

### Digital Technology and Reproductive Health Services Delivery in Africa

The application of information technology and digital tools in the delivery of sexual and reproductive health (SRH) services is gaining momentum, with the use of artificial intelligence (AI), short message service (SMS) messaging, and hotlines having been documented. For example, askNivi, an AI chatbot, was piloted in Kenya as a demand-generation tool for contraception uptake targeting adolescents and young women; it resulted in a 41% probable increase in the likelihood of contraceptive uptake among users [9]. Similarly, a study by Njagi J [10] showed that helplines (also called hotlines) provided an alternative, reliable channel for young girls and women to seek clarity and guidance on their SRH issues in a society where the nature of adult-child relations is hierarchical and conservative. The use of mobile SMS for the dissemination of health information on family planning and antenatal attendance reminders for pregnant women has been piloted in sub-Saharan Africa and has made a positive impact [11,12].

### Goal of This Study

The aim of our study was to evaluate the feasibility and acceptability of a telehealth engagement platform primarily targeting men, with behavioral and informational messaging on modern contraception (ie, family planning) and its impact on shaping their knowledge of sexual and reproductive health (SRH) and their uptake of family planning services.

## Methods

### Sample Size Calculation

The primary outcome used to estimate the study’s sample size was the change in uptake of family planning services of participants who received the men’s telehealth information package (mTIP) intervention. According to the most recent Uganda Demographic Health Survey, held in 2016, the proportion of men aged 15 to 55 that use any family planning method was 60% [2]. Assuming that there would be a 25% increase in family planning uptake due to the intervention, 5% type I error, and 90% power, we calculated that we would need to enroll 432 men. The sample size was then adjusted for an anticipated 10% attrition over the 3-month study period, which gave us a total of 475 men as the sample size to be recruited.

### Inclusion and Exclusion Criteria

Potential study participants were eligible for participation if they were aged 18 to 55 years, had a spouse or current active sexual partner (sexually active was defined as having at least 1 sexual encounter in the previous 6 months), owned a mobile phone, and were willing to take part in all study-related activities, especially periodic surveys using questionnaires. Study participants that were unable to effectively comprehend the study-related activities, unable to communicate due to suboptimal mental status or low literacy levels, or did not own a phone were excluded.

### Study Setting and Participant Recruitment

We targeted men 18 years and older who consented to participate in the study. The participant recruitment took place in 8 community settings that included academic institutions, workplaces, and social gatherings, such as at sports grounds. The study team set up a tent at each site with an appropriate level of privacy and confidentiality for the purposes of the informed consent process. Study participants that consented to take part in the study were then required to send a trigger message to the study’s SMS prepaid short code so as to start receiving mobile messages on family planning. The SMS short code was operated and maintained at The Medical Concierge Group (TMCG), a digital health and telemedicine company headquartered in Uganda that was one of the study partners. Prior to full study enrollment, a beta-test study with 25 participants was performed, with the findings used to improve the data collection tools and informed consent documents, which were resubmitted to our institutional review board for approval.

### Mobile Message Design and Dissemination

The information-behavior-motivation (IBM) skills model formed the basis of the development of the mobile messages. This model has been utilized in behavioral change approaches with the end goal of influencing adoption of positive behavior through providing correct information for informed decision-making and creating an environment that motivates the adoption of this positive behavior, for example, through reminders and nudges [13,14]. Messages on SRH focusing on modern contraception were developed by the study team and reviewed by a community advisory board and an SRH specialist for appropriateness, relevance, and local context. The messages covered IBM aspects of contraception and family planning communication. The messages were designed to go out on a weekly schedule via a prepaid short code (8884) with an average of 2 messages received weekly over a period of 60 days by the study participants. The messages were delivered in English.

### Study Participant Follow-up and Engagement

All study participants had access to a study toll-free telephone and SMS platform that was available 24 hours a day and staffed by qualified health professionals to offer remote resolutions to participants’ inquiries, including referrals and links to SRH and other health services. In addition, proactive follow up was performed by the study team at months 1, 4, and 6 after the date of study enrollment to perform specific study procedures and assess the participants for the knowledge they had gained on modern contraceptive methods, couple communication on family planning, and partner uptake of family planning.

### Data Collection

During the scheduled routine follow-up phone calls, the study participants were interviewed by one of the study staff, who was trained in phone interviews, at TMCG. During the interviews, the study team interacted with the participants to assess their awareness of family planning methods, the men’s attitudes and practices, self and spousal use of family planning, spousal communication about family planning decision-making, and the men’s opinions about their roles in family planning decision-making. The interview dates and times were negotiated and agreed upon by both the study staff and participants. The interviews were conducted in either the local language (Luganda) or English. A pretested electronic questionnaire built on an open data kit was used to collect information on the participants’ experiences with the telehealth platform and phone ownership. The study telehealth platforms (ie, SMS and the hotline) were analyzed for performance on message delivery, study participants’ engagement in quizzes, and completion of all study requirements. In-person short interviews were conducted with 25 randomly selected study participants (15 men and 10 women) to gather insights on the feasibility and accessibility of the mTIP intervention.

### Data Analysis and Interpretation

Quantitative data collected through the open data kit were analyzed using Stata software (StataCorp). Quantitative data were collected through TMCG’s telehealth platforms (SMS and the hotline) following the dissemination of the information on family planning via mobile phone. This focused on the number of SMS messages and voice calls, number of referrals, number of participants who completed all study assessments, and any other data regarding family planning, which were summarized and used as a measure of feasibility and scalability. For a qualitative inquiry, the study used in-depth interviews to elicit information from 15 men and 10 women on their experiences regarding family planning. The audio data were transcribed, coded, and thematically analyzed to address the objectives of the study. Multiple data sources from in-depth interviews with both male and female participants were used for data triangulation. We also used an information-rich description of the findings to ensure transparency. Table 1 summarizes the demographic characteristics of the interview participants.

**Table 1:** Summary of characteristics of the participants who underwent in-depth interviews.

No.	Age, years	Sex	Occupation	Marital status	Children, n	Religion	Level of education
1	56	Male	Unemployed	Married	8	Anglican	University
2	27	Male	Businessperson	Single	0	Catholic	University
3	32	Male	Teacher	Married	3	Born again	University
4	20	Male	Student	Married	0	Born again	University
5	24	Male	Driver	Married	1	Catholic	Primary
6	28	Male	Private employee	Married	2	Muslim	Secondary
7	30	Female	Private employee	Married	2	Born again	Secondary
8	25	Female	Businessperson	Married	1	Born again	Secondary
9	22	Female	Farmer	Married	2	Anglican	Secondary
10	30	Female	Casual laborer	Married	2	Catholic	Primary
11	29	Female	Teacher	Married	2	Catholic	Tertiary
12	29	Female	Housewife	Married	3	Catholic	No school
13	50	Male	Civil servant	Married	0	Seventh-day Adventist	University
14	41	Male	Religious leader	Married	5	Seventh-day Adventist	Tertiary
15	24	Male	Student	Single	0	Seventh-day Adventist	University
16	25	Male	Casual laborer	Married	1	Born again	Secondary
17	30	Male	Self employed	Married	3	Catholic	University
18	38	Male	Police officer	Married	4	Anglican	University
19	30	Male	Teacher	Married	1	Catholic	Tertiary
20	38	Male	Former security	Married	4	Catholic	Primary
21	35	Male	Teacher	Married	4	Catholic	University
22	26	Female	Businessperson	Married	2	Born again	Secondary
23	28	Female	Private employee	Married	0	Anglican	University
24	23	Female	Unemployed	Married	1	Unknown	No school
25	32	Female	Teacher	Married	6	Seventh-day Adventist	Tertiary

### Ethics Approval

The study was approved by the Joint Clinical Research Centre institutional review board (approval number 0906-2019) and registered with the Uganda National Council of Science and Technology (reference number HS425ES). All the study procedures, compensation, benefits, potential risk of participation, and the voluntary and confidential nature of participation were discussed. Written informed consent was obtained from all respondents before enrollment in this qualitative study. For young adults with low literacy, we used a thumbprint in the presence of a witness.

## Results

### Demographic Characteristics

A total of 551 study participants were recruited, including 450 men (the primary study participants) and 101 women (their spouses), who were proactively observed by the study team over a 6-month period via voice follow-up calls at months 1, 4, and 6 after enrollment. The demographic characteristics of the study participants are summarized in Table 2.

**Table 2:** Demographic characteristics of the study participants.

Variable		Men (N=450)	Spouses (N=101)
Age (years), median (IQR)		25 (22-30)	25 (23-28)
**Marital status, n (%)**			
	Single	257 (57.1)	15 (14.9)
	Married (religious, civil, or customary)	177 (39.3)	59 (58.4)
	Widowed, separated, or divorced	8 (1.8)	17 (16.8)
	Has spouse, but not legally married	8 (1.8)	10 (9.9)
**Current occupation, n (%)**			
	Student	143 (31.8)	15 (14.9)
	Employed	296 (65.8)	59 (58.4)
	Unemployed	7 (1.6)	17 (16.8)
	Declined to answer	4 (0.9)	10 (9.9)
**Education level, n (%)**			
	No education (did not complete any education)	2 (0.4)	2 (2)
	Primary level	62 (13.8)	9 (8.9)
	Secondary level	234 (52)	60 (59.4)
	University^a^	149 (33.1)	29 (28.7)
	Declined to answer	3 (0.7)	1 (1)
**Type of digital device owned (multiple choice), n (%)**			
	Basic mobile phone	281 (60.8)	39 (36.1)
	Smartphone	177 (38.3)	69 (63.9)
	Desktop computer	2 (0.4)	0 (0)
	Laptop	1 (0.2)	0 (0)
	Tablet	1 (0.2)	0 (0)

^a^Makerere and Kyambogo Universities are both non–faith-based tertiary institutions.

### User Statistics

**Figure 1:**
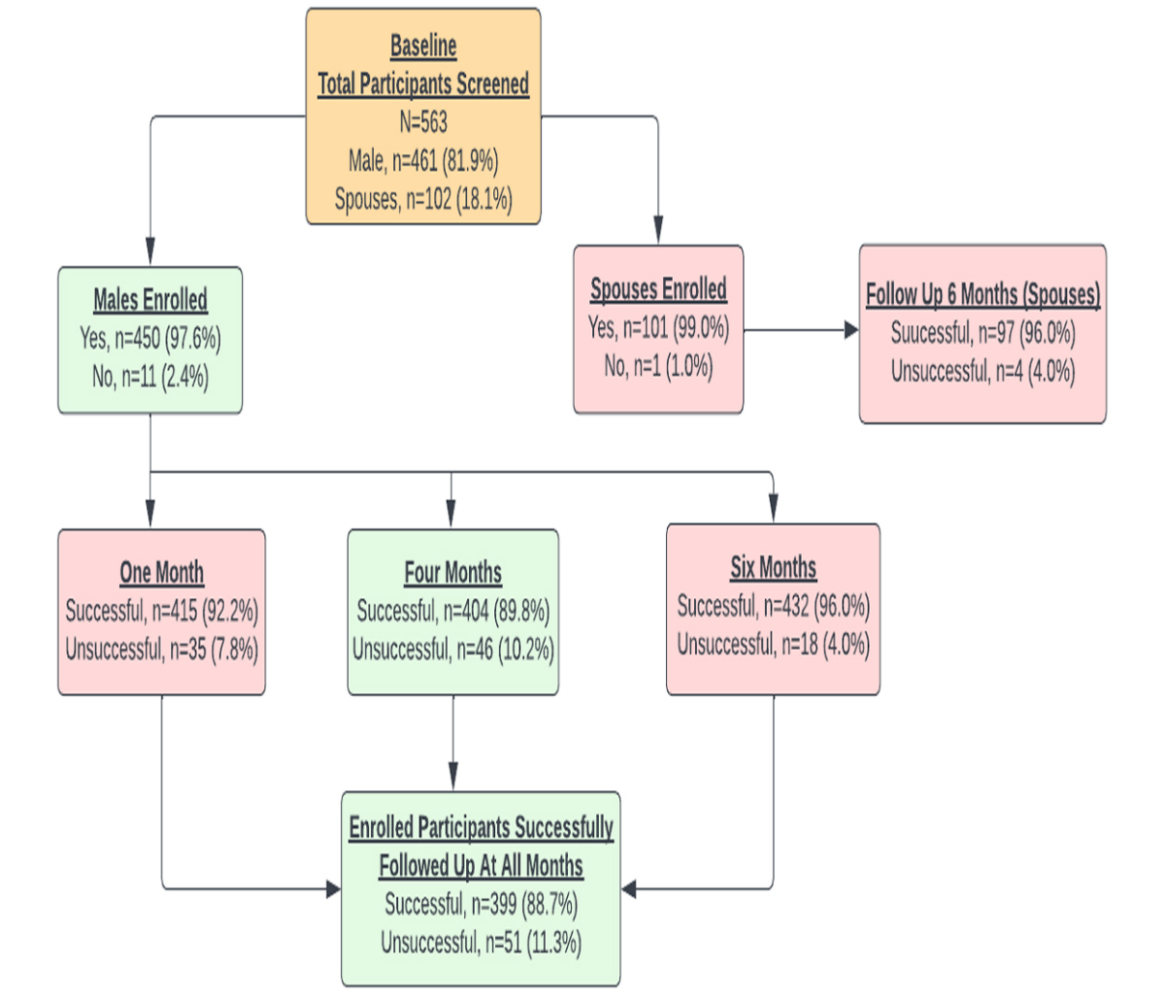
Retention Rates of Study Participants Over the 6 Months Period.

The study participants were observed for 6 months with follow-up voice calls placed 1, 4, and 6 months after the date of enrollment to assess key study-related outcomes on knowledge transfer and acquisition related to modern contraception, partner communication, and spousal uptake of family planning. The retention rate of the study participants over the 6-month period is shown in Figure 1.

### Measures of Study Participant Engagement

A total of 26,988 SMS messages were sent out over the 6-month study period, with an average of 66 messages received by each study participant. Out of the 450 men (the primary study participants) onboarded into the messaging system, 426 (95%) successfully received the messages and only 24 (5%) reported having not received them. The messages were interrupted with periodic quizzes to assess knowledge transfer and acquisition, using a total of 9 questions sent out on a weekly basis. The average response (ie, participation) rate in the weekly quizzes was 23%. We noted an 18.1% increase in couple communication attributed to the mTIP, as the couples opened up to each other more on matters concerning family planning, as highlighted in the sample responses below.

### Study Participants’ Preferences

Interview findings revealed that the men’s telehealth information package was effective and as such, feasible and acceptable for empowerment regarding family planning. As Participant 3 affirmed, “When you read these messages, you find directions...you are guided...the person cares.”

The messages were also timely, clear, educational, captivating, and laden with wise counsel, building confidence regarding family planning. They were also shareable, making it possible for the men to share the information. As Participant 3 affirmed, “It has given us confidence about family planning...when you get information from medical personnel...you are in a comfortable position to practice it.”

Additionally, the questions within the messages were not only stimulatory, but also enhanced discussion and reflection to deepen understanding. Further, the language of the messages was commended for its simplicity and clarity, accentuating access to family planning. Notwithstanding this, the respondents emphasized the need to have the messages translated into the local language to increase access, especially for those that may not be able to read and write.

The improvement in couple communication stemming from this study made it plausible for the spouses to open up to each other, rather than secretly take up family planning. The couples had candid conversations on family size, spacing, finances, and their family planning options. As Participant 1 explained, “this kind of study has enabled us...[to] come together as partners. You know in our local setting; we don’t want to share this kind of information with our wives...so this...has enabled us [to] realign our education about family planning.”

The men were empowered to support their wives regarding family planning, creating harmony in the home. Some men were relieved that their wives were using family planning, which according to Participants 9’s narrative was a relief from the potential financial strain of a large family: “I started family planning, now my second born is two years and he [her husband] sees it as very good. He is going to plan for them well, and that space is enough for a child to grow well without falling sick all the time...he is happy.”

## Discussion

**Male Involvement in Family Planning**

Innovations addressing male involvement in SRH and family planning services have mainly centered on structural barriers by extending clinic hours, allocating specific clinic times for men, and using male champions, among other strategies [13,14]. However, the need to target men with informational and behavioral messages on family planning by leveraging channels that reach them where they are located (eg, homes, workplaces, or bars) is still new, with digital solutions taking center stage [14,15].

Our study assessed the acceptability and feasibility of an mTIP that leveraged a toll-free hotline and SMS messages as channels to disseminate information on SRH and modern contraception. The 95% success rate for mobile message dissemination (426 of the 450 men successfully received the messages) shows the potential digital platforms have as effective channels for cascading family planning information to target audiences. This is especially important in reaching men who are often left out from traditional physical and mass media campaigns, as these operate in spaces that are largely seen as spaces for women, require lengthy contact time, and are not customized to meet the individual needs of men [16,17].

The 6-month retention rate of study participants in the virtual cohort was 399/450 (88.7%), positioning digital channels like SMS and voice calls as effective and sustainable platforms for continuous engagement beyond physical locations. This is supported by the rising number of people in Uganda who own mobile phones; that number stood at approximately 26 million in December 2020 [18]. In addition, the virtual cohort offered an opportunity for follow up beyond the confines of the health facility or community outreach activities, which are the traditional entry points to accessing family planning services in Uganda.

The relatively high mobile phone ownership rate among the spouses (65/101, 63.9%) offered an opportunity to diversify the digital innovations that can be deployed in the space of family planning. For example, gamified mobile applications that assess decision-making skills and knowledge transfer have been noted to stimulate more engagement with users [19,20]. The 23% participation rate of study participants in the weekly quizzes was relatively low even when compared to other studies that leveraged SMS quizzes during end user assessments. For example, the “Text-to-Change” study, which used SMS messages to disseminate information targeted at youth on HIV and AIDs, had a 53% average participation rate [21]. The discrepancy might be attributable to the absence in our study of complementary media platforms, such as flyers or a radio campaign, to increase awareness and boost participation.

Using simple, rather than complex, language or terminology in developing mobile health messages is important for end users to easily interpret the messages. This was revealed through interviews with the participants, who commended the simple, comprehensible family planning messages. In a setting where health care delivery models leave men out, owing to their work schedules and negative health-seeking behaviors, innovations that engage them within their comfort zones will be instrumental in overcoming barriers to health care access embedded within patriarchal societies in sub-Saharan Africa. This is especially important in our context, where women have traditionally sought for permission and support from their partners, in the form of transport and time, in order to access SRH services such as family planning [22,23].

In addition, given the limited contact time and space and the inadequate customization of traditional media and interpersonal communication models to suit specific local demographics for health information, current trends in mobile phone ownership in Uganda [20] offer the opportunity to leverage these ubiquitous tools for health information dissemination and reach larger audiences with minimal investment.

### Principal Results

Out of the 450 men (the primary study participants) onboarded onto the family planning mobile message plan, 426 (95%) successfully received the messages and only 24 reported having not received them. The average response (ie, participation) rate in the weekly quizzes was 23%. There was a noted 18.1% increase in couple communication attributed to the mTIP, and the couples opened up to each other more on matters concerning family planning.

### Limitations

Periodic outages of the SMS system inhibited the receipt of some of the scheduled family planning messages, disrupting information access flow. This was addressed by setting up an alert system for outages that enabled the software developers to be notified early enough for quick resolution with minimal disruption. Additionally, the unavailability of some of the study participants’ phones during the scheduled phone calls disrupted communication. This was addressed by rescheduling the follow-up call on an alternative day within the follow-up window. The study achieved 95% (551 of 576) of its target sample size, which was slightly deficient, but only negligibly affected the statistical power of the results. Notwithstanding this, the study will serve as a pilot study for a future large, randomized controlled trial of mobile phones as a channel for disseminating information on family planning, to truly measure the impact of digital telehealth as a channel for promoting family planning.

### Comparison With Prior Work

The use of mobile phones as a channel for disseminating information to bring about behavioral change has gained momentum in sub-Saharan Africa. The use of hotlines, SMS messages, mobile phone apps, and social media have been documented. In most cases, one or a combination of these channels is used to cascade health information to the target audiences, with the desired behavioral change outcomes being tracked. SMS messages have been extensively deployed in different public health programs for patient education and self-awareness of noncommunicable diseases, reminder systems in maternal health to promote antenatal attendance, and by health systems to strengthen the performance of health workers [22,23].

Therefore, the choice of SMS messages in our study as a channel for engaging with the study participants was closely informed by similar past programs. As a measure to curb message fatigue among recipients, past SMS interventions have limited the number of messages sent out to an average of 1 to 2 per week, similar to the approach taken in our study. A review of demographic health data on SMS-based family planning communication within low- and middle-income countries showed an uptake of about 5.4% within selected African countries [24]. This low utilization and uptake mirrors our 24% average participation in the periodic quizzes. Methods for assessing interventional impact in most studies have involved administering before and after interviews. For our study, we opted to perform interviews during the study at 1, 4, and 6 months from the enrollment date, in order to track changes in the outcome indicators. We believe this helped to rule out any possible impact from confounding factors that could have arisen from one-time assessment surveys or interviews.

### Conclusions

Digitally supported communications channels (SMS messages and phone hotlines) for disseminating health information on family planning could be leveraged for a wider reach with minimal resource input given limited contact time and space and the capacity for customization of the message to specific demographics. Digitally supported communication channels can provide ways to address participants’ concerns and inquiries in real time, or as fast as possible, increasing the likelihood of adoption of family planning among couples. There is a need for additional studies on the influence of mobile messaging on behavioral changes.

## Abbreviations

AI: artificial intelligence
IBM: information-behavior-motivation
mTIP: Men’s Telehealth Information Package
SMS: short message service
SRH: sexual and reproductive health
TMCG: The Medical Concierge Group
